# Determinants of help-seeking behavior in depression: a cross-sectional study

**DOI:** 10.1186/s12888-016-0790-0

**Published:** 2016-03-23

**Authors:** Anke M. Boerema, Annet Kleiboer, Aartjan T. F. Beekman, Kim van Zoonen, Henriëtte Dijkshoorn, Pim Cuijpers

**Affiliations:** Department of Clinical, Neuro, and Developmental Psychology, Section Clinical Psychology, Faculty of Behavioural and Movement Sciences, Vrije Universiteit Amsterdam, van der Boechorststraat 1, 1081 BT Amsterdam, The Netherlands; EMGO Institute for Health Care and Research, VU University Medical Centre, van der Boechorststraat 7, 1081 BT Amsterdam, The Netherlands; Department of Psychiatry, VU University Medical Centre, van der Boechorststraat 7, 1081 BT Amsterdam, The Netherlands; Statistics Netherlands (CBS), Henri Faasdreef 312, 2492 JT Den Haag, The Netherlands; GGD Amsterdam, Nieuwe Achtergracht 100, 1018 WT Amsterdam, The Netherlands

**Keywords:** Depression, Help-seeking, Stigma, Duration of symptoms

## Abstract

**Background:**

Although evidence-based and effective treatments are available for people with depression, a substantial number does not seek or receive help. Therefore, it is important to gain a better understanding of the reasons why people do or do not seek help. This study examined what predisposing and need factors are associated with help-seeking among people with major depression.

**Methods:**

A cross-sectional study was conducted in 102 subjects with major depression. Respondents were recruited from the general population in collaboration with three Municipal Health Services (GGD) across different regions in the Netherlands. Inclusion criteria were: being aged 18 years or older, a high score on a screening instrument for depression (K10 > 20), and a diagnosis of major depression established through the Composite International Diagnostic Interview (CIDI 2.1).

**Results:**

Of the total sample, 65 % (*n =* 66) had received help in the past six months. Results showed that respondents with a longer duration of symptoms and those with lower personal stigma were more likely to seek help. Other determinants were not significantly related to help-seeking.

**Conclusions:**

Longer duration of symptoms was found to be an important determinant of help-seeking among people with depression. It is concerning that stigma was related to less help-seeking. Knowledge and understanding of depression should be promoted in society, hopefully leading to reduced stigma and increased help-seeking.

## Background

Depression is an important public health issue [1] due to its high prevalence [2], the substantial impact on daily functioning [3], the markedly reduced quality of life in both patients and their relatives [4], and the high economic burden [5]. Effective and evidence-based treatments for depression, like psychotherapy and pharmacological treatments, are available [6]. However, many people do not receive professional care for their symptoms. Estimates of the number of people with depression that receive help range from 28 % to 60 % depending on the definition and measurement used [7, 8].

Considering the high burden of depression and the large treatment gap, it is important to identify reasons why people do or do not seek help for depression. This study examined determinants of help-seeking among people with major depression. The study was guided by Anderson’s behavioral model of health care utilization [9] which distinguishes three groups of determinants for help-seeking: 1) *Predisposing factors*: characteristics of individuals that exist prior to their illness, like age or gender; 2) *Enabling factors*: organizational factors which affect the accessibility of mental health care such as location and distribution of health care facilities. From the patients’ perspective this factor relates to knowledge about accessibility of health services and individuals’ financial situation; 3) *Need factors:* professional judgment of people’s health status (evaluated need for care) and individuals’ perspective on health, symptoms and functioning (perceived need for care) [9].

The focus of this study was on predisposing and need factors as several studies have demonstrated that these are dominant in help-seeking [10–12]. In addition, all Dutch residents have basic health insurance which covers health care costs from the general practitioner, primary care, and more specialized psychological care. Therefore, enabling factors are considered less important determinants of help-seeking behavior in the Netherlands.

With respect to ‘*predisposing factors’,* demographic factors, social structure, personality and health beliefs are considered to be important in the help-seeking process. Research has shown that people who are younger or middle aged and unmarried are more inclined to use health services for their psychological problems [10, 13]. Furthermore, intensified health care use is associated with low perceived social support [14] and personality characteristics, especially with high neuroticism [15, 16]. Other studies identified attitudinal or personal barriers such as the patients’ inability to recognize the problem, the belief that depression will abate [11], the desire to handle problems on one’s own, [10] and negative beliefs about the effectiveness of treatment [17]. While there is evidence that stigma influences help-seeking adversely, this has not received much attention in Anderson’s model [18]. Stigma refers to ‘a mark of shame, disgrace or disapproval which results in an individual being rejected, discriminated against, and excluded from participation in a number of different areas of society’ [19]. Two different types of stigma are often distinguished: personal and perceived stigma. Personal stigma is defined as peoples’ own attitude towards people with depression [20] whereas perceived stigma represents the perception of a persons’ belief about how other people think about depression [20]. Personal stigma is common in depression and related to less help-seeking [21, 22].

Regarding ‘*need factors’* in Andersons’ behavioral model [9], research has shown that people with depression are more likely to experience a need for treatment when symptoms are severe [23]. Additionally, they were more likely to use health services when they reported a long-term medical condition or physical symptoms or a comorbid anxiety or other mental disorders [24–27].

Despite emerging evidence, it remains unclear why people do or do not seek help, since there is no clear or single decision that determines if and when people seek help [28]. Therefore, it is important to examine predisposing and need factors within the same study. Using data from a general population in the Netherlands, this study examined predisposing (age, partner status, personality, loneliness, personal stigma, perceived stigma) and need factors (severity and duration of complaints, co-morbidity) that are related to help-seeking behavior among people with depression. This study is focused on actual help-seeking and not the intention to seek help. The pathway between going to a health professional and the intention to do this is ambiguous [28] and intentions for help-seeking do not ensure that people will actually seek or receive help for their problems [23, 29].

## Methods

### Participants

Subjects with major depressive disorder (MDD) were recruited in collaboration with three Municipal Health Services (GGD) across different regions in the Netherlands (Amsterdam, Zoetermeer/Leidschendam, Dordrecht/Gorinchem). Every four years, the Municipal Health Services conduct a survey, the Health Monitor, in a random sample of the adult population in the Netherlands. The survey includes questions about physical health, psychosocial health, life-style, environment and the K10, a screening questionnaire for psychological distress [30]. Subjects who completed the Health Monitor in 2012 and who scored high on the K10 were invited to participate. A clinical diagnostic interview (CIDI 2.1) was conducted to determine whether subjects met criteria for a current major depressive disorder.

Participants were eligible for inclusion if they: a) had a score of 20 or higher on the screening questionnaire (K10), b) were aged 18 years or older, c) scored positive for a current major depressive disorder or dysthymia measured with the Composite International Diagnostic Interview (CIDI 2.1) in the past six months. People with insufficient understanding of the Dutch language were excluded.

### Procedure

The study was approved by the medical ethical committee of the VUMC (nr 2011/394). Subjects who scored high on the K10 screening questionnaire, and who agreed that they could be approached for further study, were contacted by the Municipal Health Services by post and received an information letter and an informed consent form. Participants were asked to return the informed consent form to the research team at the VU Amsterdam, indicating their willingness to take part. Those that did not respond to this first invitation received one or two reminders.

Next, participants were invited by telephone for the diagnostic interview to determine depression status and were asked to complete an online questionnaire. If preferred, participants were allowed to complete the questionnaire by telephone (*n =* 14) or on paper (*n* = 1).

### Instruments

Depression and anxiety diagnoses were obtained with the CIDI 2.1 interview. Information about age, gender, partner status, psychological distress (K-10), loneliness and comorbidity with physical illness was gathered through the ‘Health Monitor’. The remaining information was obtained via the online questionnaire.

#### Help-seeking

To determine whether participants had received help for depression, a number of questions from the Trimbos/iMTA questionnaire for Costs associated with Psychiatric Illness (TiC-P) [31] were used. The first part of the Tic-P consists of questions on service use for psychiatric disorders, while the second part is focused on productivity losses [31]. The Tic-P has shown acceptable feasibility and reliability [32].

In the present study the first part of the TiC-P was used. Participants were asked if they had received help for mental health problems from a general practitioner, psychiatrist, psychologist, mental health institution, social worker, clinic for alcohol or drugs abuse, medical specialist, or if they had received day treatment for psychological problems in the past six months. Participants were considered to be “help-seeking” if they confirmed at least one contact with a mental health care provider in the past six months. Participants who did not, were considered to be “non-help-seeking”.

#### Depression and anxiety disorders

DSM-IV diagnoses were based on the life-time version of the CIDI (version 2.1), a fully structured, standardized questionnaire designed by the World Health Organization (WHO) [33]. It is used to assess mental disorders and provides diagnoses for scientific research [34]. In the present study, anxiety disorders (social anxiety disorder, panic disorder with or without agoraphobia, generalized anxiety disorder) and mood disorders (major depression and dysthymia) were assessed. The CIDI was found to be a reliable instrument [34]. A diagnosis of depression or anxiety in the past 6 months was used to determine a current episode.

All participants were interviewed by professionally trained master level psychology students who worked under supervision.

#### Determinants of help- seeking

##### Demographic information

Demographic characteristics that were used in this study were age, gender and partner status.

##### Social structure

Loneliness was measured with the Loneliness scale [35]. The Loneliness scale consists of 11 items. Participants could answer the questions with “yes”, “more or less” and “no”. A higher score indicates more loneliness. A scale reliability of .80 to .90 is reported in different studies [36]. In this study, the Cronbachs alpha was .87 for the emotional loneliness scale and .85 for the social loneliness scale.

##### Personality characteristics

Neuroticism was assessed with the neuroticism subscale of the NEO- Five Factor Inventory (NEO-FFI) [37]. The NEO-FFI measures five domains of personality: Neuroticism, Extraversion, Openness, Agreeableness and Conscientiousness.

The NEO-FFI neuroticism subscale includes 12 items which can be answered on a 5- point scale, ranging from ‘strongly disagree’ to ‘strongly agree’. Research has reported good internal consistency and test-retest reliability for the NEO-FFI [37]. The internal consistency of Neuroticism in the present study was sufficient (Cronbachs α = .77).

##### Stigma

Stigma was assessed with the Depression Stigma Scale (DSS) [20]. The DSS consists of two subscales; personal and perceived stigma. The 9 items of each subscale reflect several themes and include status of depression as an illness (‘depression is not a real medical illness’), personal control (‘people could snap out of depression if they wanted’), character (‘sign of weakness’), dangerousness of depression (‘people with depression are dangerous’) , unpredictability (‘people with depression are unpredictable’), shame (‘would not tell anyone’), avoidance (‘avoid people with depression’) and discrimination (‘not vote for politician with depression’ and ‘not employ someone with depression’) [20]. The personal stigma subscale is defined as peoples’ own attitude towards depression (e.g. ‘people with depression are dangerous’) [20]. The perceived stigma scale represents the perception of a persons’ belief about how other people think about depression (e.g. ‘most people believe that people with depression are dangerous’) [20]. The items can be valued on a 5-point likert scale ranging from strongly disagree to strongly degree, with a total score range of 0–36. A higher score indicates more stigma. Moderate to high internal consistency [38–39] and moderate test-retest reliability [20] was reported in several studies. The present study showed moderate to high internal consistency. Cronbach’s alpha was .77 for personal stigma and .80 for perceived stigma.

##### Psychological distress

The Kessler-10 (K10) is a brief psychological distress scale [30]. All questions referred to the past month. The K10 is a self-report scale consisting of 10 items that can be valued with a five value response: all of the time, most of the time, a little of the time and none of the time. A higher score indicates more distress. Research support the validity of the K10 as measurement of psychological distress [40]. The internal consistency of the K-10 in the present study was sufficient (Cronbachs α = .83).

##### Symptom duration

The duration of symptoms was examined with one question: “How long have you experienced symptoms? (in months)”.

##### Physical illness

Information about physical illness was available for the past year. Physical illness included chronical diseases or conditions like, heart conditions, a form of cancer, migraine, high blood pressure, asthma or COPD, dizziness, arthritis or eczema.

### Statistical analyses

Analyses were performed with SPSS 21.0 and a significance level of *p* < .05 was used in all analyses.

A multiple logistic regression analysis was performed to determine the association between the predictors and the dependent variable (help-seeking). The non-help-seeking group was used as the reference group. Six predictors in the analyses were categorical namely: partner status (0 = no partner, 1 = partner), severity of distress (0 = mild, 1 = moderate, 2 = severe), duration of symptoms (0 = 0–12 months, 1 = longer than a year ago), loneliness (0 = not lonely, 1 = lonely) and comorbidity with anxiety and physical illness (0 = no comorbidity, 1 = comorbidity). First, we conducted a series of univariable analyses for each predictor separately. Then we conducted a multivariable analysis in which all determinants were included using a backward selection procedure. The assumptions of the logistic regression analyses (linearity of the logit and multicollinearity) were tested and there was no indication of abnormality.

## Results

### Study sample

Information letters were sent to 1191 people who scored high on the K10.

**Fig. 1:**
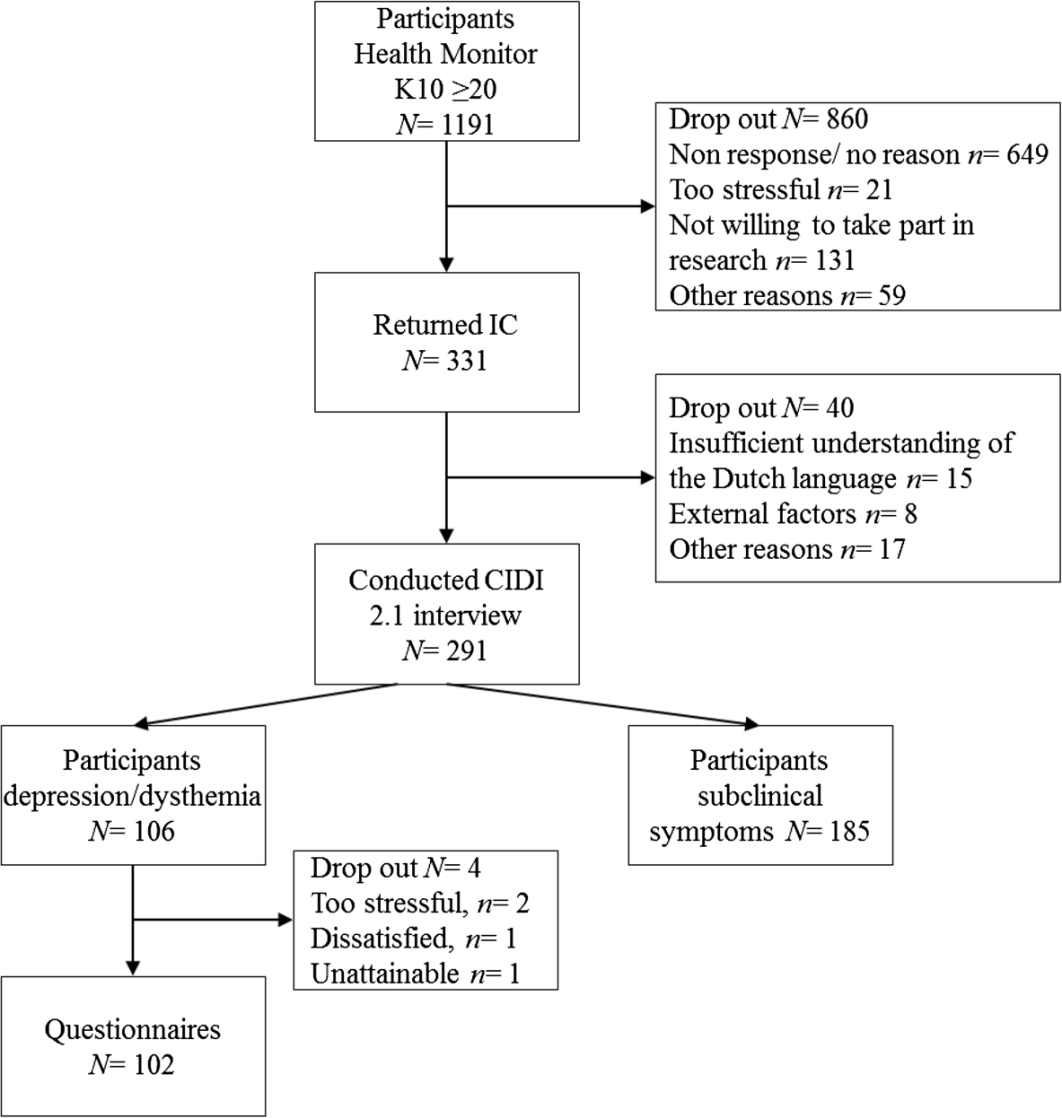
Participant flow chart

Three hundred thirty-one participants (GGD Amsterdam, *n =* 140, GGD Zuid-Holland West, *n =* 148, GGD Zuid-Holland Zuid, *n* = 43) returned their informed consent form which is a response rate of 28 %. 291 participants completed the diagnostic interview (CIDI 2.1). A sample of *n =* 106 participants met criteria for MDD. The remaining participants met criteria for subclinical depression and were not included in this paper but reported on elsewhere [41]. A final sample of *n* = 102 participants filled in the online questionnaire. Figure 1 describes the flow of participants through the recruitment process.

### Characteristics of the study sample

The sample consisted of 55 women (54 %) and 47 men (46 %). Respondents were 52 years of age on average (range 20–88). 46 % of the participants reported co-morbidity with an anxiety disorder in the past six months (*n =* 47), 78 % reported co-morbidity with physical symptoms in the past year (*n =* 77). Of the total sample, 65 % (*n =* 66) sought help in the past six months, while 35 % (*n* = 36) did not seek help. Table 1 lists the characteristics of the study sample and provides details of the participants’ diagnoses.

**Table 1:** Demographic characteristics for participants that did and did not seek help for depression

	Help-seeking (*N =* 66)	Non-help-seeking *(N =* 36)
Demographics		
Gender, *n* (%)		
Male	32 (49)	15 (42)
Female	34 (51)	21 (58)
Age, mean (SD)	51.41 (17.07)	53.26 (17.84)
Education, *n* (%)		
Low	15 (23)	10 (29)
Middle	24 (36)	11 (30)
High	27 (41)	14 (41)
Partner status, *n* (%)		
Partner	32 (49)	21 (58)
No partner	34 (51)	15 (42)
Psychological distress, *n* (%)		
Mild	20 (30)	12 (34)
Moderate	20 (30)	9 (26)
Severe	26 (40)	14 (40)
Co-morbid anxiety disorder, *n* (%)		
No	33 (50)	22 (61)
Yes	33 (50)	14 (39)
Co-morbid physical illness, *n* (%)		
No	12 (19)	5 (16)
Yes	52 (81)	27 (84)
Loneliness, *n* (%)		
No	14 (21)	5 (14)
Yes	52 (79)	30 (86)
Neuroticism, mean (SD)	27.7 (7.8)	27.0 (6.5)
Personal stigma, mean (SD)	11.8 (5.6)	15.6 (5.1)
Perceived stigma, mean (SD)	21.1 (5.1)	21.3 (5.3)
Current diagnosis, *n* (%)		
Major depression	52 (79)	30 (83)
Dysthymia	4 (6)	1 (3)
Major depression and dysthymia	10 (15)	5 (14)
Life-time diagnosis, *n* (%)		
Lifetime dysthymia, no lifetime MDD	3 (5)	0 (0)
Lifetime MDD, no lifetime dysthymia	40 (61)	23 (64)
Both lifetime MDD and lifetime dysthymia	23 (34)	13 (36)
Characteristics, mean (SD)		
Age onset first episode (8–78)	36.45 (17.43)	36.64 (18.10)
Mean episodes (1–70)	9.04 (13.42)	13.57 (16.14)
Duration of complaints, *n* (%)		
0–12 months	14 (21)	14 (41)
12 months or longer	52 (79)	20 (59)

### Help-seeking

Of the participants who had received help for psychological problems in the past six months (*n* = 66), 26 % (*n* = 17) received help in general health care (general practitioner, social work, medical specialist), 15 % (*n* = 10) received help in specialized mental health care (psychiatrist, psychologist, clinic for alcohol or drugs abuse, mental health institution, psychiatrist in hospital). The majority 59 % (*n* = 39) received help in both settings.

Univariable and multivariable logistic regression analyses were conducted to examine the association between help-seeking behavior and determinants among people with a major depressive disorder.

The univariable regression analyses showed significant odds ratios (OR) for duration of symptoms (OR = 2.60; 95 % CI = 1.05–6.41; *p* = 0.03) and personal stigma (OR = 0.89; 95 % CI = 0.83–0.96; *p* = 0.005). After univariable analyses, a backward multivariable analyses was performed to determine the effect of individual predictors controlled for each other. The final model showed significant odds ratios for duration of symptoms (OR = 2.80; 95 % CI = 1.06–7.37; *p* = 0.03) and personal stigma (OR = 0.90; 95 % CI = 0.84–0.98; *p* = 0.009). People who received treatment were more likely to experience a longer duration of symptoms and were less likely to experience personal stigma. Hosmer and Lemeshow Test indicated a good fit of the model (χ ^2^ = 6.14, *p* = 0.52, *df* = 7). Table 2 displays the results of the univariable and multivariable logistic regression analyses.

**Table 2:** Results univariable logistic model and backward logistic regression multivariable model

	Univariable			Multivariable					
				Model 1^a^			Model 2^b^		
	OR	95 % CI	*p*	OR	95 % CI	*p*	OR	95 % CI	*p*
Age	0.99	0.97–1.01	0.42	0.99	0.97–1.01	0.42			
Partner status	1.48	0.65–3.37	0.34	0.76	0.27–2.17	0.61			
Severity complaints(1)	1.33	0.46–3.86	0.59	1.19	0.31–.4.48	0.79			
Severity complaints(2)	1.11	0.42–2.92	0.82	0.92	0.24–3.45	0.90			
Duration complaints	2.60^c^	1.05–6.41	0.03^*^	2.59	0.80–8.43	0.11	2.80	1.06–7.37	0.03^*^
Comorbidity anxiety	1.57	0.68–3.58	0.28	1.28	0.38–4.28	0.68			
Comorbidity physical	0.80	0.25–2.51	0.70	0.96	0.25–3.78	0.96			
Neuroticism	1.01	0.95–1.07	0.63	0.98	0.90–1.07	0.98			
Loneliness	0.61	0.20–1.88	0.39	0.59	0.15–2.36	0.45			
Personal stigma	0.89^d, e^	0.83–0.96	0.005^*^	0.91	0.83–0.99	0.04^*^	0.90	0.84–0.98	0.009^*^
Perceived stigma	0.99	0.91–1.07	0.85	0.98	0.88–1.09	0.77			

Model (χ 2 = 11.92, *p* = 0.009, *df* = 2). *Note.* OR = odds ratio, CI = confidence interval, *Significant at .05

^a^Model 1 represents the first model

^b^Model 2 represents the final model

^c^( χ 2 = 4.31, *p* = 0.03, *df* = 1)

^d^Controlled for three residuals that seem to influence the model

^e^(χ 2 = 6.39, *p* = 0.01, *df* = 1)

## Discussion

The aim of this study was to examine determinants of help-seeking behavior among people with depression by investigating predisposing (age, partner status, personality, loneliness, personal stigma and perceived stigma) and need factors (severity and duration of symptoms, co-morbidity with anxiety or physical illness).

The main finding that duration of symptoms was associated with increased health care utilization, is consistent with previous research [11, 23]. This finding suggests that people seek help when their symptoms persist for a longer period of time. This is not necessarily an undesirable outcome, as previous research in first onset depressed patients from the community [42] has shown that 50 % remitted within 3 months. This supports the idea of watchful waiting in those with a first episode of depression and a short duration of symptoms. However, this is probably less suitable for people with a chronic or recurrent depression who are at higher risk for long-term impairments and negative consequences from depression [43]. In addition, research has shown that recovery rates for depression declined rapidly after 3 months [42], supporting the idea that long treatment delays may be harmful to patients. More research in people with a first onset of depression and a recurrent form of depression is necessary to investigate the optimal time to seek professional help.

Other need factors, like severity of symptoms and comorbid anxiety, were not related to help-seeking whereas other studies suggest that these factors increase service use in people with depression [24–27]. The direction of the results in the present study are in line with previous findings, however, we may lack statistical power due to a small sample size. Another potential reason why we did not find a relationship between help-seeking and other need factors is that we recruited a group with relatively high comorbidity regarding medical illness and anxiety disorders, suggesting that the need of treatment in both groups is relatively high.

In line with earlier research findings, we found a negative association between personal stigma and help-seeking [29]. In addition, perceived stigma was not associated with help-seeking [44, 45]. There is some evidence that personal stigma is more important than perceived stigma regarding help-seeking and that these two types of stigma should be considered as separate concepts [29, 40, 46]. A small association between personal stigma and patient’s preference to deal with depression alone was found [22]. This suggests that people with higher personal stigma, may be more inclined to handle problems by themselves and are, therefore, less likely to seek professional help.

Other predisposing factors were not related to help-seeking. These results contradict previous research that showed that younger people, those who experience more loneliness, those scoring higher on neuroticism and people who live without a partner are more inclined to use health services [12, 14, 16, 17]. This may, again, be explained by the high comorbidity in our sample. There is some evidence that illness severity is a prompt reason to seek help [11] meaning that in people with high comorbidity, predisposing factors may be less important than need factors. Furthermore, there was little difference between the help-seeking and non-help-seeking group with respect to predisposing factors. In addition, the majority of people in both the help-seeking and non-help seeking group experienced loneliness, suggesting that other factors than predisposing or need factors are important in this particular group, like for example previous experiences with help-seeking.

This study has several strengths and limitations that need to be considered when interpreting the results. We have recruited participants from a random sample in the general population, which is a strength of this study. However, the participation rate was relatively low, 28 %. Furthermore, not all respondents to the Health Monitor gave permission to be contacted for further research which may have led to an additional selection bias. We do not have information on subjects who did not want to participate in the Health Monitor or this study. However, compared to other studies (NEMESIS) [47], our sample was relatively old, had a chronic course of depression and high comorbidity. Therefore, they may have other questions, ideas and expectations concerning help-seeking than people with a first episode of depression, who are often younger and have less comorbidity. For example, research has shown that people with chronic depression have more questions on how to prevent another episode, while people with a first time episode ask for more information about how to cope with depression in daily life [48]. Furthermore, people with a chronic depression may be more demoralized and have negative expectations about the outcome of treatment, which may influence their willingness to seek help [48]. Although the nature of our sample can be considered a limitation in some respect, depression is known to have a recurrent and chronic nature [42, 49] and there is not much research that examines reasons to seek help among people with chronic depression. Another limitation of this study was the relatively small sample size which may have limited the statistical power. A final limitation of this study was the cross-sectional design, meaning that no conclusions about causality can be drawn.

## Conclusions

This study suggests that duration of symptoms and personal stigma are associated with help-seeking behavior among people with depression. Previous research has shown that depression is less stigmatized compared with other mental health disorders like schizophrenia [50]. However, this study showed that personal stigma is also related to help-seeking behavior among people with depression. More attention is needed to increase awareness about symptoms of depression to reduce personal stigma. Stigma interventions, such as educational programs, are associated with a small but significant reduction of personal stigma [20, 51]. Hopefully, such programs will lead to increased knowledge and more understanding of depression and subsequently increased help-seeking behavior.

## Availability of data and materials

Data available on request.
